# Evaluating the link between insulin resistance and cognitive impairment using estimated glucose disposal rate in a non-diabetic aging population: results from the CHARLS

**DOI:** 10.3389/fmed.2025.1522028

**Published:** 2025-06-05

**Authors:** Bingqing Wang, Fei Xu, Minheng Zhang

**Affiliations:** ^1^Department of Neurology, Taiyuan Central Hospital, Taiyuan, Shanxi, China; ^2^Department of Gerontology, The First People’s Hospital of Jinzhong, Jinzhong, Shanxi, China

**Keywords:** cognitive impairment, estimated glucose disposal rate, insulin resistance, diabetes mellitus, CHARLS

## Abstract

**Background:**

Emerging evidence suggests insulin resistance may contribute to neurodegeneration, yet its role in non-diabetic populations remains unclear. This study explores the relationship between estimated glucose disposal rate (eGDR), a measure of insulin sensitivity, and incident cognitive dysfunction in non-diabetic adults.

**Methods:**

Our longitudinal analysis utilized data from 5,178 CHARLS participants (age ≥ 45 years). Insulin sensitivity was quantified using eGDR, calculated from waist circumference, hypertension status, and hemoglobin A1c levels. Participants were stratified by eGDR quartiles for comparative analysis. We employed multivariable Cox models, survival curves, restricted cubic splines, and sensitivity testing to evaluate associations with cognitive outcomes.

**Results:**

Over an 8.7-year follow-up, cognitive dysfunction developed in 36.9% of participants. Analyses revealed significant metabolic-cognitive associations, with each standard deviation increase in eGDR linked to a 15.8% reduction in risk (adjusted hazard ratio [HR] = 0.792, 95% confidence interval [CI]: 0.793–0.881). Restricted cubic spline analysis identified non-linear threshold effects, with risk accelerating below certain eGDR levels (*P* < 0.05). Kaplan-Meier survival analysis demonstrated significant differences in cognitive impairment incidence across eGDR quartiles (*P* = 0.003). Additionally, both eGDR and metabolic score for insulin resistance (METS-IR) showed comparable predictive value for cognitive impairment risk, outperforming other metabolic indices, including the atherogenic index of plasma (AIP), and the triglyceride glucose index (TyG).

**Conclusion:**

These findings position eGDR as a promising biomarker for cognitive risk stratification in non-diabetic adults. However, further multi-database studies should validate these associations and explore the underlying mechanisms.

## Introduction

The rising prevalence of cognitive impairment poses a significant public health burden, intensified by shifting age demographics worldwide. This complex condition arises from an interplay of hereditary factors, environmental influences, and lifestyle variables. Of particular interest is insulin resistance (IR), which has gained attention as a modifiable factor linked to progressive cognitive decline (1–3). Although traditionally viewed through the lens of metabolic disease and Type 2 diabetes mellitus (T2DM), contemporary research establishes IR as an independent predictor of cognitive dysfunction even in individuals with normal glucose regulation (3–6). These findings align with insulin’s diverse neurological functions, including its involvement in brain energy homeostasis, synaptic maintenance, and neuroprotective mechanisms. Mounting evidence further implicates disrupted insulin pathways in the development of Alzheimer’s pathology and other neurodegenerative disorders.

Current diagnostic approaches for IR evaluation, which primarily rely on fasting blood glucose (FBG) and hemoglobin A1c (HbA1c) measurements, demonstrate diminished reliability in non-diabetic populations (7–10). Such metrics often fail to detect early metabolic disturbances occurring outside pancreatic regulation. eGDR, a novel composite index combining abdominal obesity, hypertensive status, and glycemic control parameters, presents a more robust solution. Prior investigations have primarily concentrated on diabetic subjects, potentially obscuring IR’s true effects through glucose-related confounding variables while also facing sample size limitations. Importantly, this innovative measure shows superior accuracy in detecting metabolic dysfunction among populations with preserved glucose tolerance (11, 12) and effectively forecasts cardiovascular-metabolic disease trajectories (13–15). Nevertheless, the connection between eGDR and cognitive performance remains unexplored. Clarifying this relationship may provide valuable tools for identifying high-risk subgroups and implementing timely preventive measures in metabolically vulnerable, non-diabetic individuals.

Utilizing the China Health and Retirement Longitudinal Study (CHARLS) dataset, this research examines how eGDR correlates with newly developed cognitive dysfunction in non-diabetic individuals. Additionally, the analysis compares the eGDR with three contemporary metabolic markers: the metabolic score for insulin resistance (METS-IR), the atherogenic index of plasma (AIP), and the triglyceride glucose index (TyG), in order to assess their respective prognostic capacities for predicting cognitive impairment. These investigations seek to clarify the role of insulin resistance and lipid metabolism in cognitive aging while establishing potential diagnostic applications for eGDR in metabolically at-risk, non-diabetic cohorts.

## Materials and methods

### Study population

**FIGURE 1:**
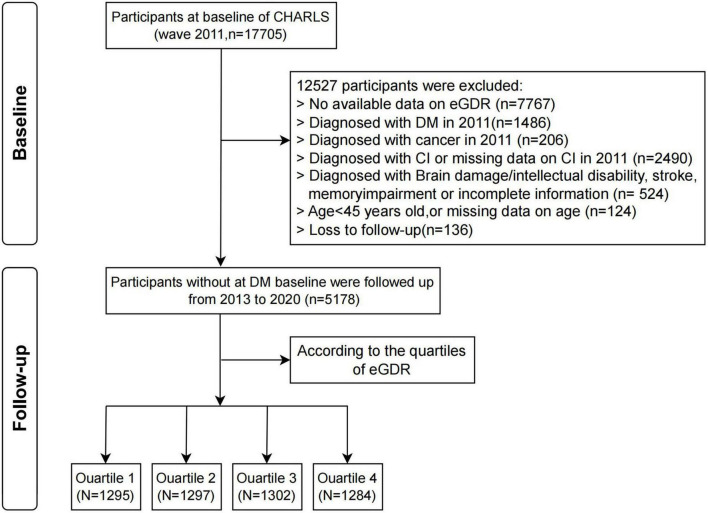
Participant selection process flowchart. eGDR, estimated glucose disposal rate; CI, cognitive impairment; CHARLS, China Health and Retirement Longitudinal Study; DM, diabetes mellitus.

This study draws upon data from the CHARLS, a nationally representative cohort study initiated in 2011, with subsequent follow-up waves in 2013, 2015, 2018, and 2020 (16). A total of 12,527 participants were excluded based on the following criteria: missing data on eGDR (*n* = 7,767); a diagnosis of DM in 2011 (*n* = 1,486); a history of brain injury, intellectual disability, stroke, or memory impairment, or incomplete information (*n* = 524); a diagnosis of cognitive impairment or missing cognitive impairment data in 2011 (*n* = 2,490); age under 45 years (*n* = 124); or loss to follow-up (*n* = 136). Following these exclusions, the final sample comprised 5,178 eligible participants (Figure 1). All study participants provided written informed consent before enrollment. This research project received ethical approval from Peking University’s Biomedical Ethics Review Committee (IRB00001052-11015), with data collection strictly limited to consenting individuals for final analysis.

### Calculation of eGDR and IR stratification

The estimated glucose disposal rate was derived from the equation: eGDR (mg/kg/min) = 21.158-(0.09 × waist circumference [cm])-(3.407 × hypertension [1 = yes, 0 = no])-(0.551 × hemoglobin A1c) [%]). Participants were then stratified by eGDR quartiles for insulin resistance level comparisons.

### Cognitive function assessment in CHARLS

The CHARLS employed the Mini-Mental State Examination (MMSE) to measure cognitive performance, utilizing this standardized tool’s capacity to evaluate both global functioning and specific domains including memory retention and cognitive processing. For memory assessment, researchers administered a ten-item verbal recall test, with participants required to repeat words both immediately following presentation and after a 5-min delay, where one point was allocated for each accurate response (potential score: 0–20). The evaluation of fundamental cognitive capacities incorporated three components: arithmetic tasks involving successive subtraction from 100, geometric figure replication to assess spatial reasoning, and temporal awareness questions regarding date identification. Performance on these measures contributed equally to a maximum of 11 points. By aggregating results from both domains (total possible: 31 points), investigators identified cognitive impairment using a validated cutoff of <11 points (17, 18).

### Potential covariates

This investigation expanded upon existing literature by incorporating a multidimensional array of covariates spanning sociodemographic attributes, health behaviors, and clinical biomarkers. Participant profiles captured age, sex, residential classification (urban/rural), geographical location (northern/southern China), educational background (categorized as ≤9 years, 10–12 years, or ≥13 years of schooling), and partnership status (married/cohabiting versus single/divorced/widowed). Health behavior indicators documented tobacco use, alcohol consumption, and sensory impairments, alongside psychosocial factors (social engagement levels and depressive symptoms) and cardiometabolic risk markers (elevated blood pressure and adiposity). Biochemical analyses quantified glycemic control (HbA1c, fasting blood glucose [FBG]), hematologic parameters (hemoglobin), and lipid profiles (total cholesterol [TC], triglycerides [TG], high-density lipoprotein cholesterol [HDL-C], low-density lipoprotein cholesterol[LDL-C]). Adiposity was determined via body mass index (BMI) (weight[kg]/height[m]^2^), classifying obesity at ≥28 kg/m^2^. Hypertension criteria included: (1) clinical diagnosis, (2) antihypertensive medication use, or (3) systolic/diastolic pressures exceeding 140/90 mmHg. Diabetes mellitus was operationalized through: (1) self-reported diagnosis, (2) glucose-lowering drug use, (3) FPG ≥ 126 mg/dL (7.0 mmol/L), or 4) HbA1c ≥ 6.5%. Depressive symptomatology was evaluated using the CESD-10 instrument (score range: 0–30 points).

### Statistical analysis

Comparisons across eGDR quartiles were conducted to examine variations in demographic, health, and metabolic characteristics, including age, sex, education, marital status, rural residence, geographic region, BMI, WC, systolic blood pressure (SBP), diastolic blood pressure (DBP), obesity, smoking, alcohol use, hemoglobin, FBG, vision impairment, HbA1c, TC, TG, HDL, LDL, diabetes, hearing loss, depressive symptoms, and social isolation. Continuous data following normal distributions were summarized as means with standard deviations (mean ± SD) and compared using parametric analysis of variance, while non-normally distributed measures were reported as medians with interquartile ranges [median (IQR)] and analyzed through non-parametric Kruskal-Wallis tests. Categorical data were expressed as frequency counts with percentages [n (%)], with group differences examined via χ^2^ tests. The dose-response association between eGDR and cognitive impairment was investigated using restricted cubic splines (RCS), with Cox proportional hazards models applied to evaluate this relationship through both continuous and categorical parameterizations of eGDR. Three progressively adjusted models were constructed: a crude model (unadjusted), a partially adjusted model (controlling for demographic factors including age, sex, residence location, marital status, and education level, along with behavioral covariates of smoking and alcohol consumption), and a fully adjusted model (incorporating all potential confounders). Additional stratified analyses were performed using multivariable Cox regression to identify potential effect modifications across population subgroups. Kaplan-Meier survival analysis with log-rank tests compared cognitive impairment risk across eGDR quartiles. Sensitivity analyses were conducted under four conditions: (1) excluding participants with cognitive impairment onset by 2013 and (2) redefining diabetes based solely on FBG and HbA1c levels.

## Results

### Baseline characteristics

Table 1 displays the baseline characteristics of participants stratified by quartiles of eGDR. Significant differences were observed across most demographic and health variables among the eGDR quartiles (*P* < 0.05). Participants in the lowest quartile of eGDR (Quartile 1, indicating higher insulin resistance) were generally older, had higher waist circumference, HbA1c, FBG, BMI, and blood pressure levels compared to those in higher eGDR quartiles. Conversely, HDL levels were lowest and triglyceride levels highest in Quartile 1, indicative of poorer metabolic health in this group. Notably, gender, vision and hearing impairment, educational level, alcohol consumption, and social isolation did not vary significantly across eGDR quartiles (*P* > 0.05).

**TABLE 1:** Baseline characteristics of participants stratified by quartiles of eGDR.

Characteristic	Quartiles of eGDR				*P*-value
	**Quartile 1**	**Quartile 2**	**Quartile 3**	**Quartile 4**	
Participants	1295	1297	1302	1284	
eGDR	9.45 ± 2.07	6.50 ± 0.68	8.92 ± 0.91	10.70 ± 0.27	<0.001
TyG	4.64 ± 0.29	4.74 ± 0.29	4.68 ± 0.29	4.61 ± 0.28	
AIP	−0.02 ± 0.31	0.09 ± 0.30	0.02 ± 0.32	−0.05 ± 0.30	
METS-IR	35.31 ± 7.58	40.39 ± 7.23	36.75 ± 7.91	34.45 ± 5.46	
Age, years	59.01 (8.42)	58.34 (8.84)	55.95 (7.90)	56.34 (8.23)	<0.001
Gender					0.257
Male	645 (49.85%)	655 (50.54%)	689 (52.92%)	679 (52.96%)	
Female	649 (50.15%)	641 (49.46%)	613 (47.08%)	603 (47.04%)	
Rural residence					<0.001
Rural	700 (54.05%)	791 (60.99%)	847 (65.05%)	901 (70.17%)	
Urban	595 (45.95%)	506 (39.01%)	455 (34.95%)	383 (29.83%)	
Region					<0.001
South	574 (44.32%)	669 (51.58%)	678 (52.07%)	802 (62.46%)	
North	721 (55.68%)	628 (48.42%)	624 (47.93%)	482 (37.54%)	
Marital status					0.002
Married and living with spouse	1105 (85.33%)	1119 (86.28%)	1172 (90.02%)	1104 (85.98%)	
Others	190 (14.67%)	178 (13.72%)	130 (9.98%)	180 (14.02%)	
Education					0.216
Junior high school and below	1122 (86.64%)	1140 (87.90%)	1120 (86.02%)	1103 (85.90%)	
Senior high school	144 (11.12%)	135 (10.41%)	165 (12.67%)	151 (11.76%)	
Junior college or above	29 (2.24%)	22 (1.70%)	17 (1.31%)	30 (2.34%)	
Smoking status					0.036
Yes	511 (39.46%)	537 (41.40%)	553 (42.47%)	578 (45.02%)	
No	784 (60.54%)	760 (58.60%)	749 (57.53%)	706 (54.98%)	
Drinking status					0.959
Yes	561 (43.35%)	570 (43.95%)	562 (43.16%)	551 (42.91%)	
No	733 (56.65%)	727 (56.05%)	740 (56.84%)	733 (57.09%)	
Blind or partially blind					0.194
Yes	58 (4.48%)	60 (4.63%)	47 (3.61%)	69 (5.37%)	
No	1237 (95.52%)	1237 (95.37%)	1255 (96.39%)	1215 (94.63%)	
Deaf or partially deaf					0.151
Yes	90 (6.95%)	78 (6.02%)	63 (4.84%)	74 (5.76%)	
No	1205 (93.05%)	1218 (93.98%)	1239 (95.16%)	1210 (94.24%)	
Obesity					<0.001
Yes	338 (26.24%)	204 (15.81%)	33 (2.54%)	8 (0.63%)	
No	950 (73.76%)	1086 (84.19%)	1264 (97.46%)	1269 (99.37%)	
Depression					0.003
Yes	382 (30.25%)	368 (29.21%)	385 (30.20%)	444 (35.46%)	
No	881 (69.75%)	892 (70.79%)	890 (69.80%)	808 (64.54%)	
Social isolation					0.171
Yes	763 (58.92%)	775 (59.75%)	779 (59.83%)	808 (62.93%)	
No	532 (41.08%)	522 (40.25%)	523 (40.17%)	476 (37.07%)	
SBP, mmHg	146.85 (19.48)	132.90 (20.39)	118.00 (11.20)	116.31 (11.58)	<0.001
DBP, mmHg	85.11 (11.67)	77.99 (11.58)	70.74 (8.70)	69.18 (8.77)	<0.001
BMI, Kg/m2	26.18 (3.39)	24.27 (3.82)	23.33 (2.51)	20.68 (2.43)	<0.001
WC, cm	93.55 (7.44)	87.41 (10.72)	84.89 (3.79)	74.95 (5.16)	<0.001
HbA1c, %	5.15 (0.41)	5.13 (0.41)	5.10 (0.36)	4.95 (0.38)	<0.001
FBG, mg/dL	103.49 (16.36)	102.47 (16.73)	100.08 (13.94)	97.67 (13.12)	<0.001
Hemoglobin, g/dL	14.78 (2.26)	14.59 (2.18)	14.43 (2.19)	14.17 (2.05)	<0.001
TC, mg/dL	199.07 (38.81)	192.80 (35.85)	191.22 (36.75)	185.21 (35.45)	<0.001
TG, mg/dL	125.67 (89.39–178.99)	109.74 (78.76–160.18)	99.12 (72.57–139.83)	85.85 (63.72–119.47)	<0.001
HDL-C, mg/dL	47.07 (13.02)	50.24 (14.66)	51.95 (14.74)	55.90 (15.18)	<0.001
LDL-C, mg/dL	122.57 (35.63)	115.98 (33.40)	116.66 (33.29)	110.91 (31.98)	<0.001

BMI, body mass index; SBP systolic blood pressure; DBP diastolic blood pressure; eGDR estimated glucose disposal rate; METS-IR, metabolic score for insulin resistance; AIP, atherogenic index of plasma; TyG, triglyceride glucose index; FBG, fasting blood glucose; HbA1c, hemoglobin A1c; TC, total cholesterol; TG, triglyceride; HDL-C, high-density lipoprotein cholesterol; LDL-C, low-density lipoprotein cholesterol; WC waist circumference; CI, cognitive impairment.

### Association between baseline eGDR and cognitive impairment incidence

**FIGURE 2:**
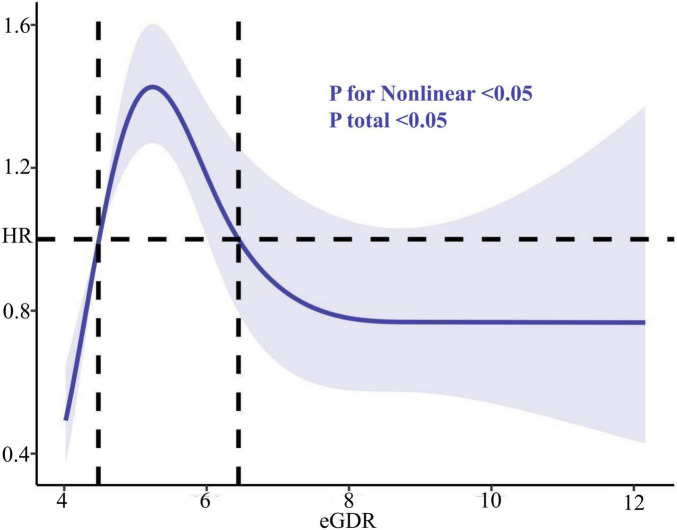
Association of eGDR and the risk of cognitive impairment using a multivariable-adjusted restricted cubic spines model. Restricted cubic spline analysis has four knots at the 5th, 35th, 65th, and 95th percentiles of eGDR. eGDR, estimated glucose disposal rate.

During the follow-up period, 1,913 participants (36.94%) developed cognitive impairment (Supplementary Table 1). The RCS analysis (Figure 2) revealed a significant non-linear association between eGDR and cognitive impairment incidence, with a higher risk of cognitive impairment observed as eGDR decreased (indicating increased insulin resistance) (*P* < 0.05). This association persisted across all adjusted models, suggesting a potential threshold effect in the link between eGDR and cognitive impairment risk.

### Cox proportional hazards and Kaplan-Meier survival analysis of the association between eGDR and cognitive impairment

**FIGURE 3:**
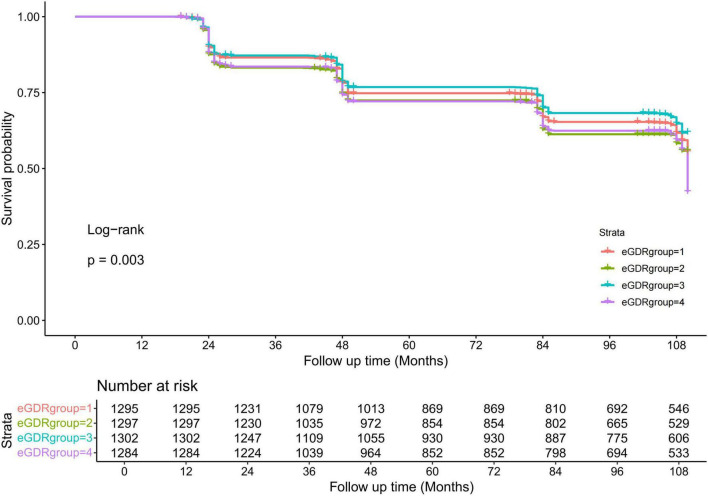
The Kaplan-Meier analysis for cognitive impairment was based on eGDR quartiles. eGDR, estimated glucose disposal rate.

The Cox proportional hazards models demonstrated an inverse relationship between eGDR and cognitive impairment risk. Progressive multivariable adjustment revealed consistent associations: each unit reduction in eGDR corresponded to a 21.8% lower risk (HR = 0.792, 95%CI: 0.745–0.801, *P* = 0.014) in the unadjusted model, 19.5% (hazard ratio [HR] = 0.805, 95% confidence interval [CI]: 0.795–0.818, *P* = 0.014) after demographic adjustment, and 15.8% (HR = 0.842, 95%CI: 0.793–0.881, *P* = 0.039) in the fully-adjusted model (Table 2). When analyzed categorically, the highest three eGDR quartiles showed non-significant protective trends (all HR < 1, *P* > 0.05) in Model III (Table 2). Supporting these findings, Kaplan-Meier curves displayed significant divergence in cognitive impairment incidence by eGDR quartile (log-rank *P* = 0.003), with progressively shorter median survival times observed in lower quartiles (Figure 3).

**TABLE 2:** Multivariate-adjusted hazard ratios (95% confidence intervals) of estimated glucose disposal rate for cognitive impairment.

eGDR	Total N	No. of cognitive impairment	Model 1		Model 2		Model 3	
			**HR (95%CI)**	***P*-value**	**HR (95%CI)**	***P*-value**	**HR (95%CI)**	***P*-value**
**Continues**								
**Per SD increase**	5178	1913 (36.94)	0.792 (0.745, 0.801)	0.014	0.805 (0.795, 0.818)	0.018	0.842 (0.793, 0.881)	0.039
**Quartiles**								
Q1	1295	462 (35.68)	Reference		Reference		Reference	
Q2	1297	509 (39.24)	0.830 (0.797, 0.862)	0.057	0.835 (0.781, 0.866)	0.068	0.842 (0.823, 0.867)	0.114
Q3	1302	441 (33.87)	0.811 (0.779, 0.838)	0.163	0.823 (0.808, 0.841)	0.703	0.844 (0.824, 0.867)	0.486
Q4	1284	501 (39.02)	0.826 (0.791, 0.853)	0.097	0.803 (0.768, 0.839)	0.005	0.823 (0.795, 0.846)	0.051

HR, hazard ratio; CI, confidence interval; eGDR, estimated glucose disposal rate. Model1: unadjusted; Model 2: adjusted for age, sex, rural residence, marital status, education level, smoking status and drinking status; Model 3: adjusted all confounding factors(age, sex, rural residence, marital status, education level, region, smoking status, drinking status, deaf or partially deaf, blind or partially blind).

### Cox proportional hazards models comparing METS-IR, AIP, and TyG versus eGDR for CI risk

In the fully adjusted models, three metabolic indices demonstrated distinct associations with cognitive impairment. The metabolic score for insulin resistance (METS-IR) exhibited a significant inverse relationship, with each standard deviation increase corresponding to a reduced risk of cognitive impairment (HR = 0.99, 95%CI: 0.98–1.00, *P* = 0.002) (Supplementary Table 2). This protective effect was more pronounced in the quartile analyses, where participants in the highest METS-IR quartile had an 18% lower risk of cognitive impairment compared to those in the lowest quartile (HR = 0.82, 95%CI: 0.72–0.94, *P* = 0.005) (Supplementary Table 2). For the atherogenic index of plasma (AIP), linear regression analysis revealed a non-significant trend (HR = 0.90, 95%CI: 0.78–1.05, *P* = 0.170), although participants in the highest AIP quartile approached marginal significance (HR = 0.89, 95%CI = 0.78–1.02, *P* = 0.100) (Supplementary Table 3). In contrast, the triglyceride glucose index (TyG) demonstrated a near-significant linear association with cognitive impairment risk (HR = 0.85, 95% CI: 0.72–1.00, *P* = 0.050), with participants in the highest TyG quartile showing robust protection against cognitive impairment (HR = 0.83, 95%CI: 0.73–0.95, *P* = 0.010) (Supplementary Table 4).

### Subgroup analysis

The association between eGDR and cognitive impairment risk demonstrated significant heterogeneity by smoking status. Among never-smokers, each SD increment in eGDR corresponded to a 12.2% lower risk (HR = 0.822, 95%CI: 0.784–0.861, *P* = 0.038). Smokers showed a similar but non-significant inverse relationship (*P* = 0.216), with significant between-group heterogeneity (pinteraction = 0.023). No significant effect modification was observed for age, sex, or alcohol consumption (all pinteraction > 0.05, Table 3).

**TABLE 3:** Subgroup analysis of the association between eGDR (per 1 SD) and cognitive impairment.

Variables	HR (95%CI)	*P*-value	P _interaction_
**Age, years**			0.610
<60	0.983 (0.866, 1.117)	0.792	
≥60	0.082 (0.796, 0.844)	0.639	
**Gender**			0.070
Male	0.952 (0.842, 1.077)	0.433	
Female	1.113 (0.991, 1.250)	0.071	
**Smoking status**			0.023
Yes	0.919 (0.804, 1.051)	0.216	
No	0.822 (0.784, 0.861)	0.038	
**Drinking status**			0.081
Yes	0.951 (0.835, 1.082)	0.447	
No	1.107 (0.991, 1.237)	0.072	

HR, hazard ratio; CI, confidence interval; eGDR, estimated glucose disposal rate.

### Sensitivity analysis

Sensitivity analyses using alternative modeling approaches consistently showed modest associations between continuous eGDR measurements and cognitive outcomes (Table 4). Both models produced comparable effect estimates, reinforcing the primary findings while demonstrating robustness to different analytical specifications.

**TABLE 4:** Sensitivity analysis of the association between eGDR (Q1–Q4) and cognitive impairment.

eGDR	Total N	No. of cognitive impairment	HR (95%CI)	*P*-value
**FBG+HbA1c**				
**Continues**				
**Per SD increase**	5228	1932 (36.95)	0.845 (0.832, 0.864)	0.041
**Quartiles**				
Q1	1308	467 (35.70)	Reference	
Q2	1306	512 (39.20)	0.842 (0.783, 0.905)	0.114
Q3	1307	445 (34.05)	0.877 (0.824, 0.927)	0.486
Q4	1307	508 (38.87)	0.833 (0.807, 0.864)	0.121
**Excluded CI during or before wave 2**				
**Continues**				
**Per SD increase**	3943	983 (24.93)	0.851 (0.804, 0.896)	0.033
**Quartiles**				
Q1	986	248 (25.15)	Reference	
Q2	985	251 (25.48)	0.883 (0.865, 0.901)	0.867
Q3	984	233 (23.68)	0.853 (0.748, 0.934)	0.697
Q4	988	251 (25.40)	0.868 (0.797, 0.906)	0.832

HR, hazard ratio; CI, confidence interval; eGDR, estimated glucose disposal rate; FBG, fasting blood glucose; HbA1c, hemoglobin A1c.

## Discussion

This investigation demonstrates that both the eGDR and METS-IR show similar predictive value for cognitive impairment risk, while outperforming other metabolic indices including the TyG and AIP. These results suggest that comprehensive measures of insulin sensitivity provide better prognostic capability than lipid-focused metrics for assessing cognitive risk. The comparable performance of these two insulin sensitivity markers emphasizes the fundamental role of insulin resistance in cognitive decline, consistent with their common physiological basis in glucose metabolism regulation (19–22). In contrast, the TyG displays only modest predictive ability, indicating its more limited capacity to reflect the complex metabolic dysfunction associated with neurodegeneration. Similarly, the AIP shows the weakest association, suggesting that lipid-centered evaluations offer comparatively less insight into cognitive trajectory modulation than measures of insulin-glucose homeostasis.

The role of insulin resistance in metabolic disorders is well-established, and it is now being more commonly linked to neurodegenerative processes. Studies have documented that insulin resistance adversely affects cognitive function, particularly in populations at risk for metabolic syndrome or diabetes (1, 4–6, 23–25). Reflecting the current literature, our research highlights the crucial role of insulin sensitivity in cognitive health, suggesting that eGDR may serve as a significant marker for assessing cognitive risk in individuals without diabetes. In contrast to studies that depend only on fasting glucose or HbA1c, eGDR includes extra factors such as waist size and blood pressure, giving a fuller picture of insulin resistance (26, 27). The analysis of subgroups uncovered a significant association between eGDR and cognitive impairment risk in non-smokers, whereas this was not the case for smokers, implying a potential interaction effect. Non-smokers with lower eGDR levels had a higher risk of cognitive impairment, while smokers did not exhibit this pattern. Smoking is known to exacerbate oxidative stress and vascular inflammation, which may interact with insulin resistance in complex ways, potentially diminishing the observable impact of eGDR on cognitive impairment in this subgroup (27). Future research could further elucidate the biological interactions between smoking and insulin resistance in relation to cognitive health. Significant differences in survival without cognitive impairment across eGDR quartiles were shown by the Kaplan-Meier survival analysis, with participants in higher quartiles (indicating lower insulin resistance) experiencing longer periods free from cognitive impairment. These findings underscore the cumulative impact of metabolic health on cognitive outcomes over time, reinforcing the notion that insulin sensitivity plays a protective role against cognitive decline. This aligns with studies suggesting that maintaining metabolic health can delay or prevent the onset of neurodegenerative diseases (28–31). The sensitivity analyses, which included models adjusting for various potential confounders, confirmed the robustness of our findings. The relationship between eGDR and cognitive impairment risk was stable across these models, even after redefining diabetes solely by FBG and HbA1c levels and excluding those with early cognitive decline. This research highlights eGDR’s effectiveness as a predictor of cognitive impairment risk, especially among non-diabetic groups. However, additional longitudinal studies with more refined insulin resistance measures may further strengthen these findings.

This study has several limitations. First, while we controlled for multiple confounders, unmeasured factors may still influence the observed relationships. Second, eGDR was only measured at baseline, limiting our ability to observe changes in insulin resistance over time. Furthermore, using self-reported data on health behaviors, including smoking and alcohol use, could result in biases in reporting. Lastly, the generalizability of our findings may be limited to non-diabetic populations within a specific age range, underscoring the need for studies in diverse cohorts. Future research could focus on longitudinal changes in eGDR and their relationship with cognitive outcomes, particularly in populations at risk for both metabolic and cognitive disorders. Studying the biological pathways that associate insulin resistance with cognitive impairment may also offer valuable insights into targeted interventions. Moreover, examining the interaction effects of lifestyle factors, such as smoking and dietary habits, on the insulin resistance-cognitive impairment relationship could guide more personalized preventive strategies.

## Conclusion

These findings suggest that elevated insulin resistance, as reflected by reduced eGDR levels, may represent a modifiable risk factor for cognitive decline in non-diabetic middle and older adults. The observed correlation underscores the potential of eGDR measurements in cognitive risk assessment, necessitating further research to clarify its role in predictive modeling and to inform strategies for maintaining cognitive health in aging populations.

## Data Availability

The original contributions presented in this study are included in this article/Supplementary material, further inquiries can be directed to the corresponding author.
