# Supplementary material for: Evaluating the link between insulin resistance and cognitive impairment using estimated glucose disposal rate in a non-diabetic aging population: results from the CHARLS
Source: Front Med (Lausanne). 2025 Jun 5;12:1522028. doi: 10.3389/fmed.2025.1522028 (PMC12176753; doi:10.3389/fmed.2025.1522028)
Supplement: Supplementary file 1 [file Table_1.docx]

**Supplementary Table1** Baseline characteristics of participants stratified by cognitive impairment.

| **Characteristics** | **Non-cognitive impairment** | **Cognitive impairment** | **P-value** |
| --- | --- | --- | --- |
| **Participants** | 3265 | 1913 |  |
| **eGDR** | 9.45 (2.07) | 9.45 (2.06) | 0.995 |
| **Age, years** | 55.99 (7.87) | 59.84 (8.86) | <0.001 |
| **Gender** |  |  | <0.001 |
| Male | 1777 (54.44%) | 891 (46.65%) |  |
| Female | 1487 (45.56%) | 1019 (53.35%) |  |
| **Rural residence** |  |  | <0.001 |
| Rural | 1864 (57.09%) | 1375 (71.88%) |  |
| Urban | 1401 (42.91%) | 538 (28.12%) |  |
| **Education** |  |  | <0.001 |
| Junior high school and below | 2639 (80.83%) | 1846 (96.50%) |  |
| Senior high school | 533 (16.32%) | 62 (3.24%) |  |
| Tertiary | 93 (2.85%) | 5 (0.26%) |  |
| **Marital status** |  |  | <0.001 |
| Married and living with spouse | 2914 (89.25%) | 1586 (82.91%) |  |
| Others | 351 (10.75%) | 327 (17.09%) |  |
| **Region** |  |  | <0.001 |
| South | 1502 (46.00%) | 1221 (63.83%) |  |
| North | 1763 (54.00%) | 692 (36.17%) |  |
| **Smoking** |  |  | 0.144 |
| Yes | 1399 (42.85%) | 780 (40.77%) |  |
| No | 1866 (57.15%) | 1133 (59.23%) |  |
| **Drinking status** |  |  | 0.038 |
| Yes | 1451 (44.44%) | 793 (41.47%) |  |
| No | 1814 (55.56%) | 1119 (58.53%) |  |
| Depression |  |  | <0.001 |
| Yes | 876 (27.32%) | 703 (38.14%) |  |
| No | 2331 (72.68%) | 1140 (61.86%) |  |
| **Social isolation** |  |  | <0.001 |
| Yes | 1808 (55.38%) | 1317 (68.84%) |  |
| No | 1457 (44.62%) | 596 (31.16%) |  |
| **Blind or partially blind** |  |  | <0.001 |
| Yes | 109 (3.34%) | 125 (6.53%) |  |
| No | 3156 (96.66%) | 1788 (93.47%) |  |
| **Deaf or partially deaf** |  |  | <0.001 |
| Yes | 156 (4.78%) | 149 (7.79%) |  |
| No | 3108 (95.22%) | 1764 (92.21%) |  |
| **Obesity** |  |  | 0.002 |
| Yes | 402 (12.38%) | 181 (9.51%) |  |
| No | 2846 (87.62%) | 1723 (90.49%) |  |
| **WC, cm** | 85.75 (9.77) | 84.32 (10.00) | <0.001 |
| **HbA1c, %** | 5.08 (0.40) | 5.10 (0.40) | 0.039 |
| **FBG, mg/dL** | 101.04 (15.52) | 100.76 (14.87) | 0.527 |
| **Hemoglobin, g/dL** | 14.62 (2.16) | 14.27 (2.20) | <0.001 |
| **TC, mg/dL** | 191.03 (36.53) | 193.90 (37.89) | 0.008 |
| **TG, mg/dL** | 105.32 (75.22-153.99) | 101.78 (72.57-143.59) | 0.005 |
| **HDL-C, mg/dL** | 50.62 (14.84) | 52.41 (14.57) | <0.001 |
| **LDL-C, mg/dL** | 115.95 (33.36) | 117.56 (34.64) | 0.102 |
| **SBP, mmHg** | 127.69 (19.91) | 129.94 (21.21) | <0.001 |
| **DBP, mmHg** | 76.07 (12.13) | 75.22 (11.96) | 0.015 |
| **BMI, Kg/m2** | 23.93 (3.61) | 23.09 (3.71) | <0.001 |
| **eGDRgroup** |  |  | 0.009 |
| Q1 | 833 (25.51%) | 462 (24.15%) |  |
| Q2 | 788 (24.13%) | 509 (26.61%) |  |
| Q3 | 861 (26.37%) | 441 (23.05%) |  |
| Q4 | 783 (23.98%) | 501 (26.19%) |  |

**Abbreviation:** BMI, body mass index; SBP systolic blood pressure; DBP diastolic blood pressure; eGDR estimated glucose disposal rate; FBG, fasting blood glucose; HbA1c, hemoglobin A1c; TC, Total cholesterol; TG, Triglyceride; HDL-C, High-density lipoprotein cholesterol; LDL-C, Low-density lipoprotein cholesterol; WC waist circumference

**Supplementary Table 2** Multivariate-adjusted hazard ratios (95% confidence intervals) of METS-IR for cognitive impairment.

| **METS-IR** | **Total N** | **No. of cognitive impairment** | **Model 1** | | **Model 2** | | **Model 3** | |
| --- | --- | --- | --- | --- | --- | --- | --- | --- |
|  |  |  | **HR (95% CI)** | **P value** | **HR (95% CI)** | **P value** | **HR (95% CI)** | **P-value** |
| **Continues** |  | | | | | | | |
| **Per SD increase** | 5178 | 1913 (36.94) | 0.97 (0.97,0.98) | <0.001 | 0.98 (0.98, 0.99) | <0.001 | 0.99 (0.98, 1.00) | 0.002 |
| **Quartiles** |  |  |  |  |  |  |  |  |
| Q1 | 1295 | 462 (35.68) | Reference |  | Reference |  | Reference |  |
| Q2 | 1297 | 509 (39.24) | 0.79 (0.70,0.89) | <0.001 | 0.89 (0.79, 1.01) | 0.06 | 0.93 (0.83, 1.05) | 0.270 |
| Q3 | 1302 | 441 (33.87) | 0.67 (0.59,0.76) | <0.001 | 0.80 (0.70, 0.91) | <0.001 | 0.85 (0.75, 0.97) | 0.020 |
| Q4 | 1284 | 501 (39.02) | 0.58 (0.51,0.67) | <0.001 | 0.73 (0.64, 0.83) | <0.001 | 0.82 (0.72, 0.94) | 0.005 |

HR = Hazard Ratio; CI = Confidence Interval

Model1: unadjusted; Model 2: adjusted for age, sex, rural residence, marital status, education level, smoking status and drinking status; Model 3: adjusted all confounding factors(age, sex, rural residence, marital status, education level, region, smoking status, drinking status, deaf or partially deaf, blind or partially blind )

**Abbreviation:** METS-IR, metabolic score for insulin resistance

**Supplementary Table 3** Multivariate-adjusted hazard ratios (95% confidence intervals) of AIP for cognitive impairment.

| **AIP** | **Total N** | **No. of cognitive impairment** | **Model 1** | | **Model 2** | | **Model 3** | |
| --- | --- | --- | --- | --- | --- | --- | --- | --- |
|  |  |  | **HR (95% CI)** | **P value** | **HR (95% CI)** | **P value** | **HR (95% CI)** | **P-value** |
| **Continues** |  | | | | | | | |
| **Per SD increase** | 5178 | 1913 (36.94) | 0.76 (0.66,0.88) | <0.001 | 0.86 (0.74, 1.00) | 0.040 | 0.90 (0.78, 1.05) | 0.170 |
| **Quartiles** |  |  |  |  |  |  |  |  |
| Q1 | 1295 | 462 (35.68) | Reference |  | Reference |  | Reference |  |
| Q2 | 1297 | 509 (39.24) | 0.98 (0.86,1.10) | 0.690 | 0.99 (0.87, 1.12) | 0.85 | 1.01 (0.89, 1.14) | 0.930 |
| Q3 | 1302 | 441 (33.87) | 0.88 (0.78,1.00) | 0.040 | 0.90 (0.79, 1.02) | 0.10 | 0.92 (0.81, 1.05) | 0.220 |
| Q4 | 1284 | 501 (39.02) | 0.76 (0.67,0.87) | <0.001 | 0.85 (0.74, 0.96) | 0.01 | 0.89 (0.78, 1.02) | 0.100 |

HR = Hazard Ratio; CI = Confidence Interval

Model1: unadjusted; Model 2: adjusted for age, sex, rural residence, marital status, education level, smoking status and drinking status; Model 3: adjusted all confounding factors(age, sex, rural residence, marital status, education level, region, smoking status, drinking status, deaf or partially deaf, blind or partially blind )

**Abbreviation:** AIP, atherogenic index of plasma

**Supplementary Table 4** Multivariate-adjusted hazard ratios (95% confidence intervals) of TyG for cognitive impairment.

| **TyG** | **Total N** | **No. of cognitive impairment** | **Model 1** | | **Model 2** | | **Model 3** | |
| --- | --- | --- | --- | --- | --- | --- | --- | --- |
|  |  |  | **HR (95% CI)** | **P value** | **HR (95% CI)** | **P value** | **HR (95% CI)** | **P-value** |
| **Continues** |  | | | | | | | |
| **Per SD increase** | 5178 | 1913 (36.94) | 0.81 (0.69,0.95) | 0.010 | 0.83 (0.71, 0.98) | 0.030 | 0.85 (0.72, 1.00) | 0.050 |
| **Quartiles** |  |  |  |  |  |  |  |  |
| Q1 | 1295 | 462 (35.68) | Reference |  | Reference |  | Reference |  |
| Q2 | 1297 | 509 (39.24) | 0.88 (0.77,1.00) | 0.040 | 0.88 (0.78, 1.00) | 0.060 | 0.90 (0.79, 1.02) | 0.100 |
| Q3 | 1302 | 441 (33.87) | 0.99 (0.88,1.12) | 0.900 | 0.97 (0.85, 1.09) | 0.580 | 0.98 (0.86, 1.11) | 0.720 |
| Q4 | 1284 | 501 (39.02) | 0.79 (0.70,0.90) | <0.001 | 0.81 (0.71, 0.92) | 0.001 | 0.83 (0.73, 0.95) | 0.010 |

HR = Hazard Ratio; CI = Confidence Interval

Model1: unadjusted; Model 2: adjusted for age, sex, rural residence, marital status, education level, smoking status and drinking status; Model 3: adjusted all confounding factors(age, sex, rural residence, marital status, education level, region, smoking status, drinking status, deaf or partially deaf, blind or partially blind )

**Abbreviation:** TyG, triglyceride glucose index
